# Mapping similarities in mTOR pathway perturbations in mouse lupus nephritis models and human lupus nephritis

**DOI:** 10.1186/ar2541

**Published:** 2008-11-03

**Authors:** Padmalatha S Reddy, Holly M Legault, Joseph P Sypek, Mark J Collins, Elizabeth Goad, Samuel J Goldman, Wei Liu, Stuart Murray, Andrew J Dorner, Margot O'Toole

**Affiliations:** 1 Biological Technologies, Wyeth Research, Cambridge, 35 CambridgePark Drive, Massachusetts 02140, USA; 2 Inflammation, Wyeth Research, 200 CambridgePark Drive, Cambridge, MA 02140, USA; 3 Polysaccharides Quality, Wyeth Pharmaceutical, 1 Burtt Rd, Andover, MA 01810, USA; 4 Stryker Biotech, 35 South St, Hopkinton, MA 01748, USA; 5 ajDorner Consulting, 20 Baskin Road, Lexington, MA 02421, USA

## Abstract

**Introduction:**

Treatment with sirolimus, a mammalian target of rapamycin (mTOR) inhibitor, has been shown to be efficacious in the MRL/lpr and NZB × NZW F1 mouse models of lupus nephritis, indicating a critical role for the mTOR pathway in both models. This type of demonstration of efficacy in animal models is usually a pre-requisite for advancement into clinical development. However, efficacy in an animal model often has not translated to the desired activity in the clinic. Therefore, a more profound understanding of the mechanistic similarities and differences between various animal models and human diseases is highly desirable.

**Methods:**

Transcriptional profiling was performed on kidneys from mice with lupus nephritis; from mice who had efficacious drug treatment; and from mice before they developed nephritis. Analysis of variance with false discovery rate adjusted to p < 0.05 and an average fold change of two or more was used to identify transcripts significantly associated with disease and response to therapy. Pathway analyses (using various bioinformatics tools) were carried out to understand the basis for drug efficacy in the mouse model. The relevance in human lupus of the pathways identified in the mouse model was explored using information from several databases derived from the published literature.

**Results:**

We identified a set of nephritis-associated genes in mouse kidney. Expression of the majority of these returned to asymptomatic levels on sirolimus treatment, confirming the correlation between expression levels and symptoms of nephritis. Network analysis showed that many of these nephritis genes are known to interact with the mTOR pathway. This led us to ask what human diseases are linked to the mTOR pathway. We constructed the mTOR pathway interactome consisting of proteins that interact with members of the mTOR pathway and identified a strong association between mTOR pathway genes and genes reported in the literature as being involved in human lupus.

**Conclusions:**

Our findings implicate the mTOR pathway as a critical contributor to human lupus. This broad pathway-based approach to understanding the similarities in, and differences between, animal models and human diseases may have broader utility.

## Introduction

Clinical development of therapies is heavily dependent on demonstrated efficacy in animal model(s), but efficacy in animal models often does not translate into clinical success. A number of factors have been proposed as contributing to this lack of concordance between efficacy in animal and clinical studies [1-5]. One clear limitation of relying on disease models in inbred strains is that the genes that produce the disease phenotype in a given model may represent only a subset of the genes that can cause the phenotype in complex human diseases such as lupus. Using our own animal model transcriptomics, the vast and rapidly accumulating literature on genes linked to human disease and pathway tools, we have taken a broad analytical approach to identifying similarities between the mouse and human 'lupus phenotype' at the level of biological pathway perturbations. The potential advantage of this approach is that, by linking the human disease phenotype to a pathway, drug development efforts can be targeted to the pathway. Animal models with involvement of the same pathway can then be chosen and/or derived.

Systemic lupus erythematosus (SLE) is a chronic inflammatory autoimmune disease [6-8]. The pathophysiology of disease is manifested by the production of autoantibodies directed against multiple self-antigens. This dysregulation of the immune system resulting in the loss of tolerance appears to be mediated by both T cells and B cells. Many organs including the kidney can be affected [9]. Direct action of autoantibodies, deposition of immune complexes and pro-inflammatory cytokines, particularly interferon (IFN) γ, have all been implicated in disease pathophysiology [10-13].

There are at least four mouse models of lupus nephritis [14]. Both NZB × NZW F1 [15] and MRL/lpr mouse [16,17] strains spontaneously develop autoimmune lupus nephritis. Female mice from the NZB × NZW F1 cross (NZB/W) develop proteinuria and only a small number (< 20%) survive to 52 weeks. In MLR/lpr mice, the disease develops in both males and females and is associated with the fas lpr mutation on the MLR background. Mice develop significant proteinuria at 16 weeks and show significant mortality rates (about 50%) by 20 weeks [18,19]. Despite their independent derivation, lupus nephritis in both MLR/lpr and NZB/W mouse models shows a remarkably efficacious response to sirolimus treatment [20-22].

Sirolimus (rapamycin) is an immunosuppressive drug that binds to mTOR (mammalian target of rapamycin), a serine/threonine kinase that regulates cellular proliferation and metabolism and blocks G1 to S phase cell cycle progression, interfering with T and B cell activation [23-25]. Sirolimus is approved for the prevention of transplant rejection [26]. We used our own data (presented here) and previously published data on the efficacy of mTOR inhibitors in two mouse models of lupus nephritis to infer that perturbations of the mTOR pathway are critical to the development of lupus nephritis in both these models. In order to assess the likelihood of mTOR pathway involvement in human lupus, we examined the concordance between the mTOR pathway interactome and genes linked to human lupus and report the results of this analysis here.

## Materials and methods

### NZB/W mice

NZB/W (NZB × NZW F1 cross) females were purchased from the Jackson Laboratory (Bar Harbor, ME). These mice were maintained and studied under pathogen free conditions in accordance with guidelines from the American Association for the Accreditation of Laboratory Animal Care and the Institutional Animal Care and Use Committee of Wyeth Research.

### Experimental design

Beginning at 20 weeks of age, disease progression was monitored weekly by assessing proteinuria. A cohort of mice, selected at 20 weeks of age, served as the 'asymptomatic' group (n = 4). Once fixed proteinuria of 30 to 100 mg/dL had appeared on two consecutive occasions, (generally at 25 to 29 weeks of age), the diseased mice were randomly assigned to either the sirolimus-treated group (n = 6 mice) or the untreated (disease) group (n = 6). Sirolimus, dissolved in carboxymethylcellulose (vehicle), was subcutaneously administered three times weekly in single doses of 1 mg/kg or 5 mg/kg for eight weeks. Mice were monitored weekly until 52 weeks of age.

### Assessment of proteinuria

Urine was manually expressed from each mouse on a weekly basis, collected into a sterile container and assayed for the presence of protein (specifically albumin) using a colorimetric method (Albustix Reagent Strips, Bayer Corporation, Elkhart, IN).

Proteinuria evaluations were scored as follows: grade 0.5 = 'trace' proteinuria; grade 1 = about 30 mg/dL; grade 2 = about 100 mg/dL; grade 3 = about 300 mg/dL; and grade 4 = more than 2000 mg/dL. If mice achieved a grade 4 reading on two consecutive days they were euthanised.

### Assessment of renal pathology

Kidneys were harvested from mice one to four months after an eight-week course of treatment. Three to five mice were examined in each group. One-half of a kidney was fixed by overnight immersion in 10% formaldehyde and paraffin-embedded. The other half was snap frozen for RNA preparation. To determine the extent of renal damage, sections were stained with H & E and periodic acid-Schiff (PAS) and scored for pathological changes. In addition, glomerulopathy was scored on a 0 to 5 scale. Severity grades were as follows: 0 = normal or within normal limits; 1 = minimal or slight; 2 = mild; 3 = moderate; 4 = marked; 5 = severe.

### RNA purification and microarray hybridisation

Snap frozen murine kidney tissue was homogenised in RLT buffer containing 1% beta-mercaptoethanol using the polytron and RNA purified by Qiagen RNeasy columns (Qiagen, Valencia, CA). Eluted RNA was quantified using a Spectramax 96-well plate UV reader (Molecular Devices, Sunnyvale, CA, USA) monitoring A260/280 OD values. The quality of each RNA sample was assessed by capillary electrophoresis alongside an RNA molecular weight ladder on the Agilent 2100 bioanalyser (Agilent Technologies, Palo Alto, CA, USA).

### Microarray processing

Five micrograms of total kidney RNA was prepared from individual NZB/W F1 mice of the following groups; untreated mice at 12 weeks (n = 4); untreated F1 mice at 36 and 42 weeks combined (n = 6); and sirolimus-treated mice at 36 and 42 weeks combined (n = 6). The animals selected for expression analysis reported here were representative of multiple studies performed that confirmed the data on proteinuria, mortality rates and histopathology here.

Biotin-labelled cRNA was prepared using an oligo T7-primed reverse transcription reaction followed by *in vitro *transcription reaction with biotin-labelled UTP and CTP. cRNA 15 μg was fragmented and hybridised to Mu11KsubA and Mu11KsubB arrays (Affymetrix, Santa Clara, CA). Hybridised arrays were washed and stained with Streptavidin R-phycoerythrin (Molecular Probes Inc., Eugene, OR) using the GeneChip Fluidics Station 400 (Affymetrix, Santa Clara, CA) and scanned with a Hewlett Packard GeneArray Scanner (Hewlett Packard Company, Palo Alto, CA) according to the manufacturer's protocols. All array images were visually inspected for defects and quality. Arrays with excessive background, low signal intensity or major defects within the array were eliminated from further analysis. GeneChip MAS 5.0 software was used to evaluate the hybridisation intensity, compute the signal value for each probe set and make an absent/present call. GeneChip signal data for the samples analysed in this study are available under Accession Number [GEO:GSE12924] [27].

### Data normalisation and filtering

GeneChips were required to pass standardised quality control criteria. RNA quality was monitored by the ratio of frequencies measured by independent probe sets representing 5' and 3' regions of glyceraldehyde 3-phosphate dehydrogenase. This ratio must be more than 0.4. Filtering criteria for individual probe sets required that a probe set was called 'present' or a signal of 50 or more in at least one of the samples. All filtering criteria were passed by 6384 probe sets and were subject to the statistical analysis described below, and probe sets that did not meet these criteria were not included in subsequent analyses.

### Hierarchical clustering

For hierarchical clustering of probe sets and arrays, the Log-2 scale MAS5 expression values from each probe set were first z-normalised so each probe set had a mean expression level of zero and a standard deviation (SD) of one across all samples. Then these normalised profiles were clustered hierarchically using an unweighted paired group method with arithmetic mean, and the Euclidean distance measure.

### Identification of genes associated with lupus nephritis and response to sirolimus therapy

The disease-related fold change differences were calculated by determining the difference in the log-2 signal of the 12-week-old asymptomatic mice and the combined 36 and 42-week-old diseased mice. Analysis of variance (ANOVA) was performed using this metric to identify disease-related differences. Raw p values were adjusted for multiplicity of testing using the false discovery rate (FDR) procedure of Reiner and colleagues [28] using Spotfire (Somerville, MA). Genes with a FDR p < 0.05 and an absolute fold change of two of more in the comparison between disease and asymptomatic groups were identified as being significantly associated with lupus nephritis. Lupus nephritis genes were identified as being significantly associated with response to sirolimus treatment if they met an FDR p < 0.05, in comparison between sirolimus-treated and disease groups. Those that failed to meet the FDR p < 0.05 in the comparison of the sirolimus-treated group to the disease group, did have a significant difference (FDR p < 0.05) in the comparison between sirolimus-treated and asymptomatic groups, confirming a resistance to sirolimus therapy.

### Pathway analysis

Pathway analysis was performed using Ingenuity Pathways Analysis (IPA) (Ingenuity Systems, Redwood City, CA), MetaCore (GeneGo Inc., St. Joseph, MI) and an in-house implementation of the sigPathway algorithm [29]. SigPathway is an algorithm that identifies differentially expressed gene sets. An FDR p < 0.01 was used to identify significantly changing gene sets. Networks were built using genes that met FDR p < 0.05 within a gene set. Networks, canonical pathways and functional processes for genes passing either the FDR p < 0.05 and an absolute fold change of two or more criteria and/or SigPathway filter FDR p < 0.05 were analysed using IPA. Human, mouse and rat lupus-associated genes were identified using the search tools within IPA and MetaCore. Rapalog-mTOR pathway and its connectivity to the lupus nephritis disease data and the lupus disease genes were explored using the pathway building tools in IPA. The rapalog-mTOR pathway interactome was built using IPA and all proteins were exported to MetaCore to explore the human disease representation on the mTOR pathway interactome. In MetaCore, a gene is considered to be associated with a condition (disease, pathological or toxic process) as a biomarker if this gene or its product (RNA or protein) has different properties in the disease and healthy states. Such properties may include DNA characteristics (eg, mutation, single nucleotide polymorphisms, chromosomal rearrangement); epigenetics (eg, methylation of a promoter region, histone acethylation, nucleosome misplacement); RNA level (eg, disease specific splice variant, higher/lower expression level); or protein level (eg, disease-specific isoform, mutant isoform, subcellular localisation, post-translational modification, abundance). These genes are used to generate human 'disease biomarkers' networks using direct interactions between biomarker genes and proteins from their MetaBase database. I2E (from Linguamatics Ltd., Cambridge, UK) and clinical database findings were used to validate some of the relationships between sirolimus analogues and various human diseases identified in the mTOR pathway interactome.

## Results

### Short course therapy with sirolimus prevents onset of murine lupus and renal damage

**Figure 1:**
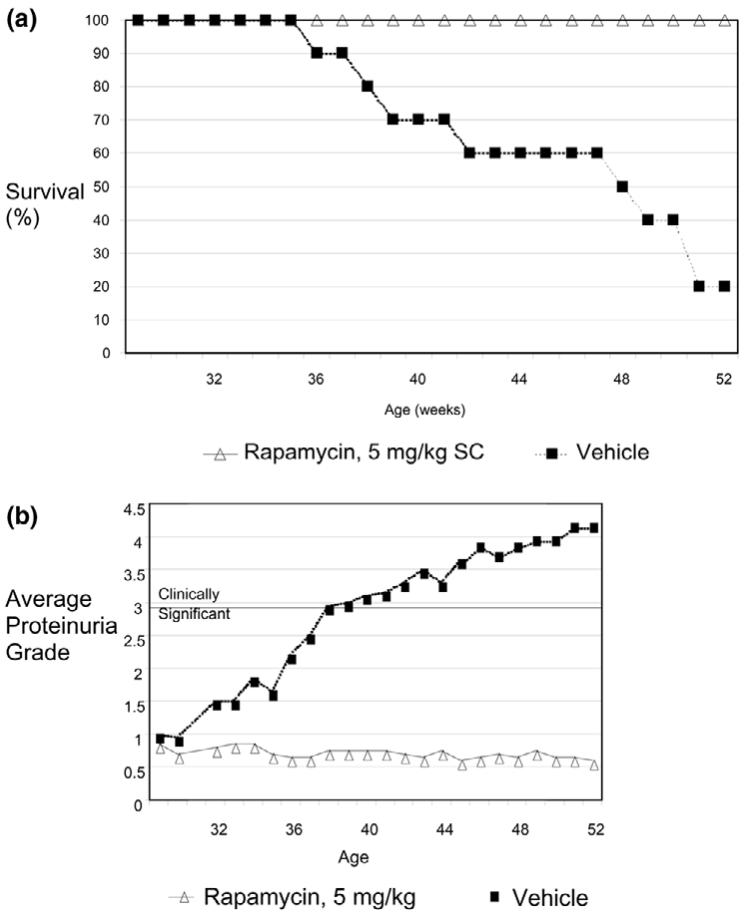
**Proteinuria and survival measurements**. (a) Survival and (b) proteinuria were measured weekly starting at 29 weeks of age (n = 10). Grade 0.5 proteinuria = 'trace'; grade 1 = about 30 mg/dL; grade 2 = about 100 mg/dL; grade 3 = about 300 mg/dL, a clinically significant level; grade 4 = more than 2000 mg/dL; grade 5 = death.

Treatment with sirolimus maintained 100% survival at age one year, although survival in the control mice was only 20% (Figure 1a). Similarly, mice treated with sirolimus had minimal or no increase in proteinuria and were asymptomatic for more than three months after cessation of treatment (Figure 1b). Collectively, these findings demonstrated the sustained benefit of a short course of sirolimus therapy initiated early in disease.

**Figure 2:**
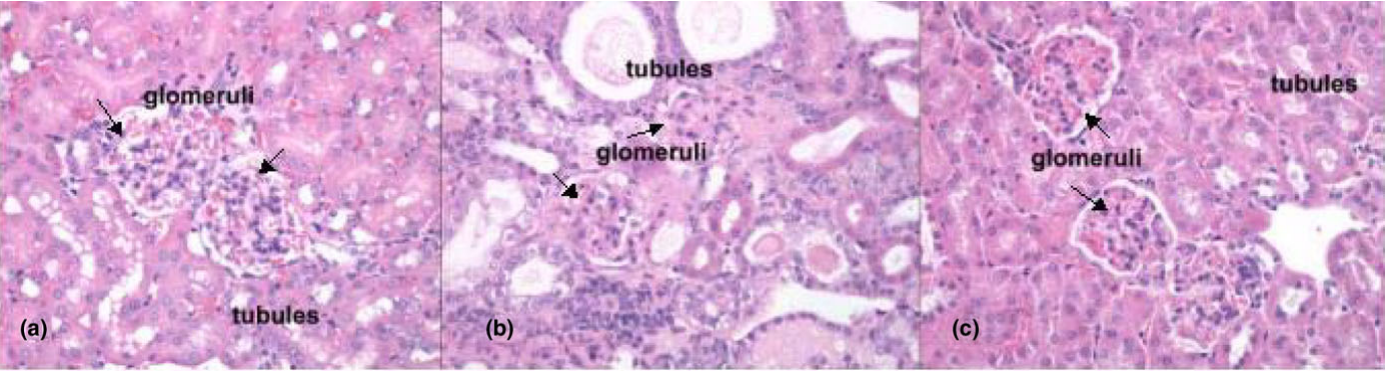
**Kidney histology. Representative sections of renal cortex from NZB × NZW F1 female mice**. (a) Section of kidney with glomeruli (arrows) and tubules that are within normal limits from a 12-week-old mouse. (b) Section from an untreated 36-week-old mouse. Glomeruli (arrows) are small with markedly thickened basement membranes and decreased numbers of cells (glomerulosclerosis). Tubules are dilated and contain protein casts or droplets in the lumens. (c) Section from a 36-week-old mouse treated with sirolimus has kidneys that are within normal limits. Glomerular (arrows) basement membranes are slightly thicker than those in the untreated 12-week-old mice. H & E, 40× original magnification.

To verify the therapeutic effects of sirolimus therapy on renal pathology, kidney tissues were evaluated microscopically for renal lesions and cellular infiltrates that were anticipated to develop in NZB/W F1 mice at 36 weeks. Light microscopy of kidney sections from vehicle-treated nephritic mice revealed glomerulonephritis and interstitial inflammation, and also proteinaceous tubular casts, consistent with their proteinuria (Figure 2b). Kidney sections from 12-week-old mice before disease onset (Figure 2a) and from 36-week-old sirolimus-treated mice (Figure 2c) revealed minimal renal pathology. There was almost a complete absence of glomerular proliferation, interstitial infiltrates and casts. Histology scores for renal inflammation, lymphocytic infiltrates and tubular atrophy are shown in Table 1. There was good correlation between the level of proteinuria and the severity of pathophysiological changes observed in the kidneys. We have also collected extensive data in this model showing a dramatic decrease in anti-dsDNA titre with sirolimus treatment (see additional file 1). Collectively, these findings confirmed the previously reported beneficial effects of sirolimus treatment on the onset and pathology of lupus nephritis in this mouse model [20,22,30].

**Table 1:** Histopathology evaluation of kidneys from NZB/W F1 hybrid female mice at 42 weeks of age

Group	Animal	Interstitial inflammation	Glomerular nephritis		Tubular dilation
				
Therapy			Cellularity	Membrane thickness	
				
None	1	+2	-2	+2	+3
None	2	+3	-1	+3	+3
None	3	+2	-1	+3	+2
None	4	+3	-3	+3	+4
None	5	+3	-3	+3	+4
None	Mean	+2.6	-2	+2.8	+3.2
					
Sirolimus*	1	(+1)	0	+1	+1
Sirolimus	2	(+1)	0	+1	0
Sirolimus	3	(+2)	0	0	0
Sirolimus	4	0	0	0	0
Sirolimus	5	(+1)	0	0	0
Sirolimus	Mean	+1	0	+0.4	0
	p value, untreated versus treated	0.004	0.002	0.00006	0.0001

0 = normal, 1 = slight, 2 = mild, 3 = moderate, 4 = marked, 5 = severe.

* denotes focal not diffuse.

### Identification of the disease-associated transcriptome

**Figure 3:**
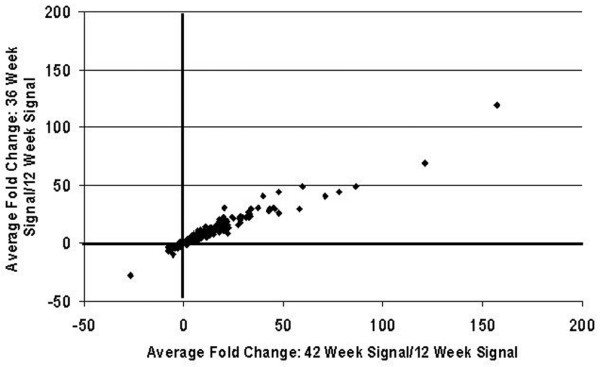
**Close similarities in disease-related gene expression changes in kidneys at 36 and 42 weeks of age**. Between the 42-week diseased group and the asymptomatic group with false discovery rate (FDR) p < 0.05 and average fold change more than 1.5, 1141 probe sets were identified to be differentially expressed. Average fold changes of these 1141 probe sets for the 42-week comparison with the asymptomatic group are shown on the X axis. The average fold changes for the 36-week comparison with the asymptomatic group are on the Y axis. Note, that although the magnitude of the changes at 42 weeks tended to be slightly larger than those at 36 weeks, the direction of change of all 1141 probe sets was the same and the magnitude of the changes are very well correlated (Pearson correlation = 0.97). The criteria used to identify 1141 probe sets are more relaxed than those used to identify lupus nephritis genes. This was to show that even the smaller changes (1.5 versus twofold change) observed in this model are well correlated at 36 weeks and 42 weeks.

RNA was prepared from tissue corresponding to one-half of a kidney containing all cortex and medullary structures and harvested from asymptomatic mice at 12 weeks, diseased mice at 36 and 42 weeks and sirolimus-treated mice at the same age. Expression levels were assessed using Affymetrix GeneChips (Affymetrix, Santa Clara, CA). There were 6384 probe sets that met the criteria for inclusion in analysis. The expression patterns of these 6384 probe sets across groups were visualised using an unsupervised clustering algorithm, which assigns samples to clusters (nodes) based on similarity of transcriptional pattern. A visual representation of differential gene expression is shown in additional file 2. Samples were grouped into three nodes: asymptomatic 12-week-old group; 36 and 42 week diseased group; and 36 and 42 week sirolimus-treated group. This segregation indicated that the renal RNA expression patterns of these three groups were distinct from each other. We then identified 1141 probe sets that differed between the asymptomatic and 42-week diseased groups with FDR p < 0.05 and an average fold change more than 1.5. As seen in Figure 3, these 1141 probe sets showed an almost identical change relative to the asymptomatic group in the comparison with the 36-week diseased group (Pearson correlation = 0.97).

**Figure 4:**
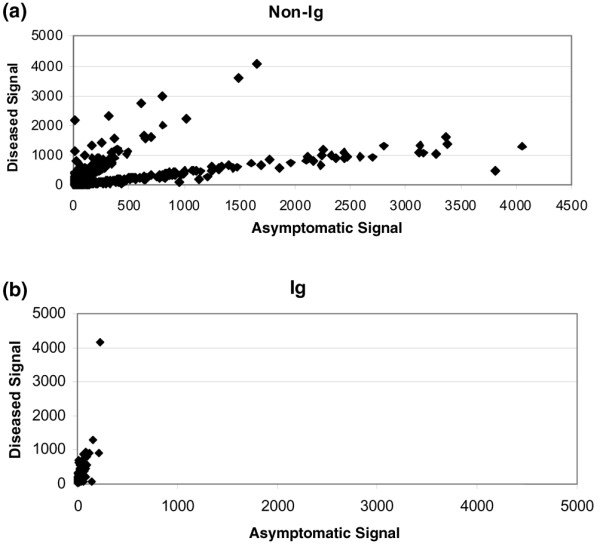
**Expression changes in transcripts associated with disease progression**. Scatter plot showing the expression levels of dysregulated probe sets expressed in (NZBxNZW)F1 kidneys of mice with lupus nephritis (older mice) (Y axis) compared with expression levels in asymptomatic (younger) mice (X axis). (a) Expression levels of 547 non-immunoglobulin (Ig) probe sets significantly associated with disease, 46% of which are expressed at higher levels in diseased kidney. (b) Expression levels of 195 immunoglobulin probes sets, 100% of which are expressed at higher levels in diseased kidney.

Based on the similarities in the 36-week and 42-week mice, ANOVA with FDR adjustment was performed comparing the expression values of the disease group (consisting of untreated 36- and 42-week-old mice) to those of the asymptomatic 12-week-old group. This analysis yielded 195 differentially expressed immunoglobulin probe sets and 547 differentially expressed non-immunoglobulin probe sets (using significance criteria of FDR p ≤ 0.05 and a fold change differential ≥ 2). The disease-associated expression pattern of the 547 non-immunoglobulin transcripts included both up-regulated and down-regulated non-immunoglobulin genes (Figure 4a). All 195 immunoglobulin probe sets were elevated in disease compared with asymptomatic animals (Figure 4b). Of these 547 probe sets, protein interaction data from the literature is available in IPA for 387 genes. We have used this set of 387 genes for pathway analyses as described below. The complete list of non-immunoglobulin genes with functional annotation is included in Additional file 3. An analysis of the expression of these genes in kidneys of young (12 weeks old) versus aged (32 weeks old) C57Bl/6 mice by ANOVA with FDR adjustment showed no significant age-related changes in the 547 transcripts associated with lupus nephritis.

### Identification of nephritis-associated probe sets modulated by sirolimus treatment

**Figure 5:**
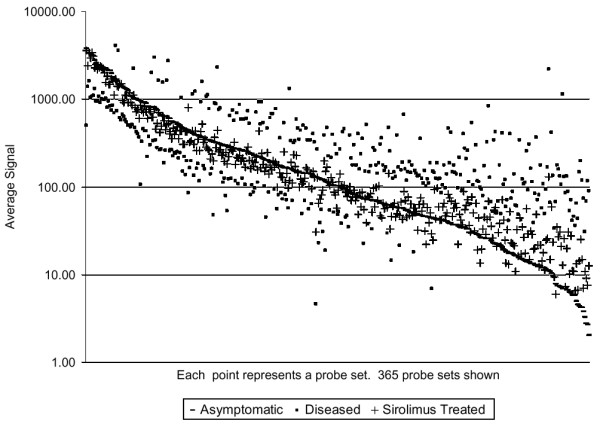
**Maintenance of normal levels of nephritis-associated gene expression with sirolimus treatment**. Of the 547 probe sets expressed at significantly different levels in diseased and asymptomatic kidney, 365 were not significantly different (false discovery rate (FDR) > 0.05) between asymptomatic and sirolimus-treated kidney. Probe sets are shown ordered by expression level in asymptomatic kidney.

Of the 547 non-immunoglobulin probe sets associated with nephritis at 36 and 42 weeks, 365 were modulated toward asymptomatic levels by sirolimus treatment (with 150 that were not modulated). Those that failed to meet the FDR p < 0.05 in the comparison of the sirolimus-treated group to the disease group, did have a significant difference (FDR p < 0.05) in the comparison between sirolimus treated and asymptomatic groups, confirming a resistance to sirolimus therapy. The comparative expression levels for the 365 sirolimus-modulated probe sets are shown in Figure 5. Both up- and down-regulated genes are among those modulated by treatment. The changes associated with therapy and amelioration of disease can be found in Additional file 3.

### Biological annotation of disease and drug-responsive genes

**Figure 6:**
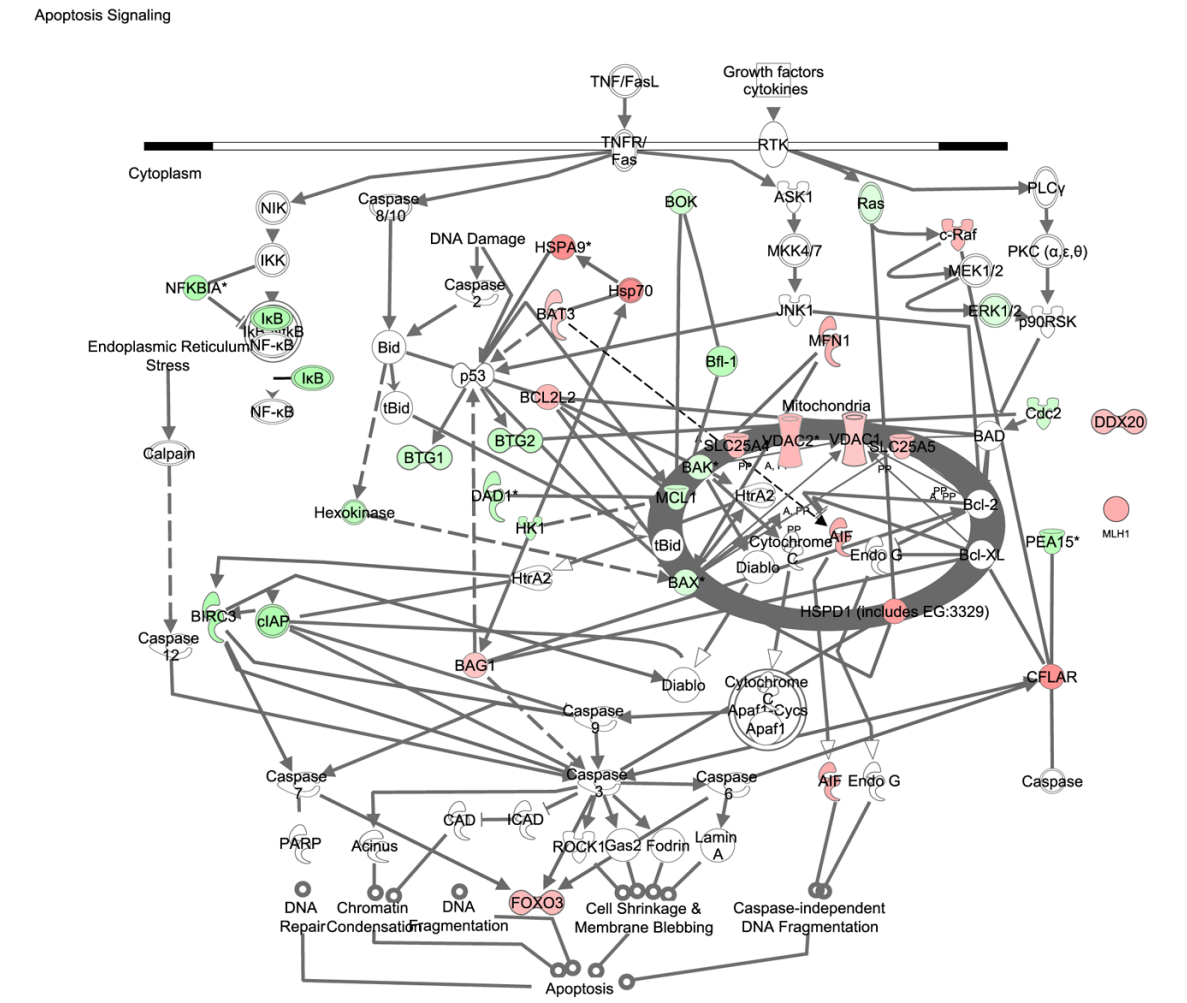
**Mitochondrial regulation of the apoptotic pathway is altered in disease and modulated by sirolimus treatment**. Expression of mitochondrial and cytosolic components involved in apoptosis in nephritis compared with asymptomatic tissue. The thick grey oval represents the mitochondria. The horizontal double line represents the plasma membrane. Two genes (DDX20 and MLH1) known to be involved in apoptosis were not connected to the main pathway in IPA. Green colour indicates higher level of expression in disease and red colour indicates lower level of expression in disease. The names of the genes are in "nodes". The shape of the nodes is related to gene function/family membership. The lines between the nodes represent the connectivity between genes. Solid lines indicate direct protein-protein interactions. Dotted lines represent indirect interactions.

**Figure 7:**
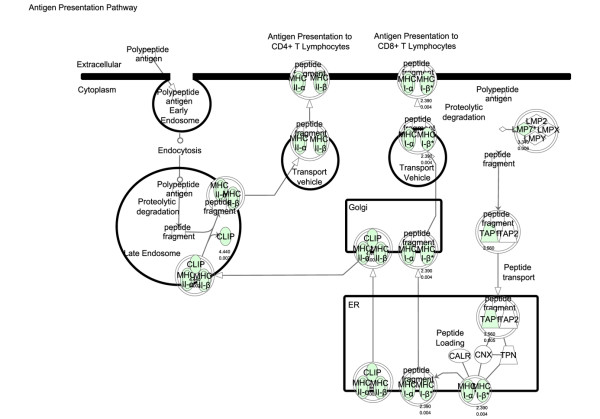
**Antigen presentation pathway altered in disease**. Expression of components of the antigen presentation pathway in nephritis compared with asymptomatic tissue. Green colour indicates higher level of expression in disease. The names of the genes are in "nodes". The shape of the nodes is related to gene function/family membership. The lines between the nodes represent the connectivity between genes. Solid lines indicate direct protein-protein interactions. Dotted lines represent indirect interactions.

**Figure 8:**
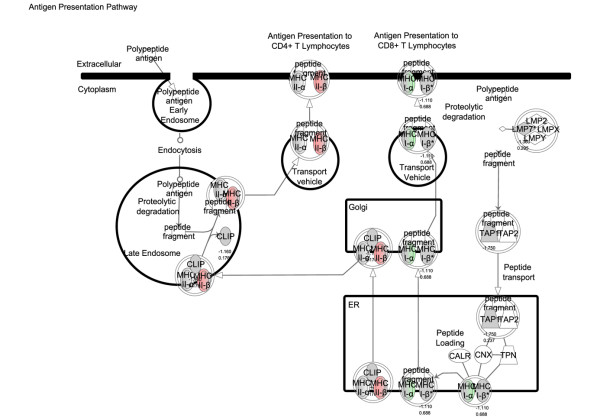
**Antigen presentation pathway modulated by sirolimus treatment**. Expression of components of the antigen presentation pathway in sirolimus-treated tissue compared with asymptomatic tissue. Green colour indicates higher level of expression in sirolimus-treated tissue and red colour indicates lower level of expression in sirolimus-treated tissue. Grey colour indicates no significant difference in expression level. The names of the genes are in "nodes". The shape of the nodes is related to gene function/family membership. The lines between the nodes represent the connectivity between genes. Solid lines indicate direct protein-protein interactions. Dotted lines represent indirect interactions.

**Figure 9:**
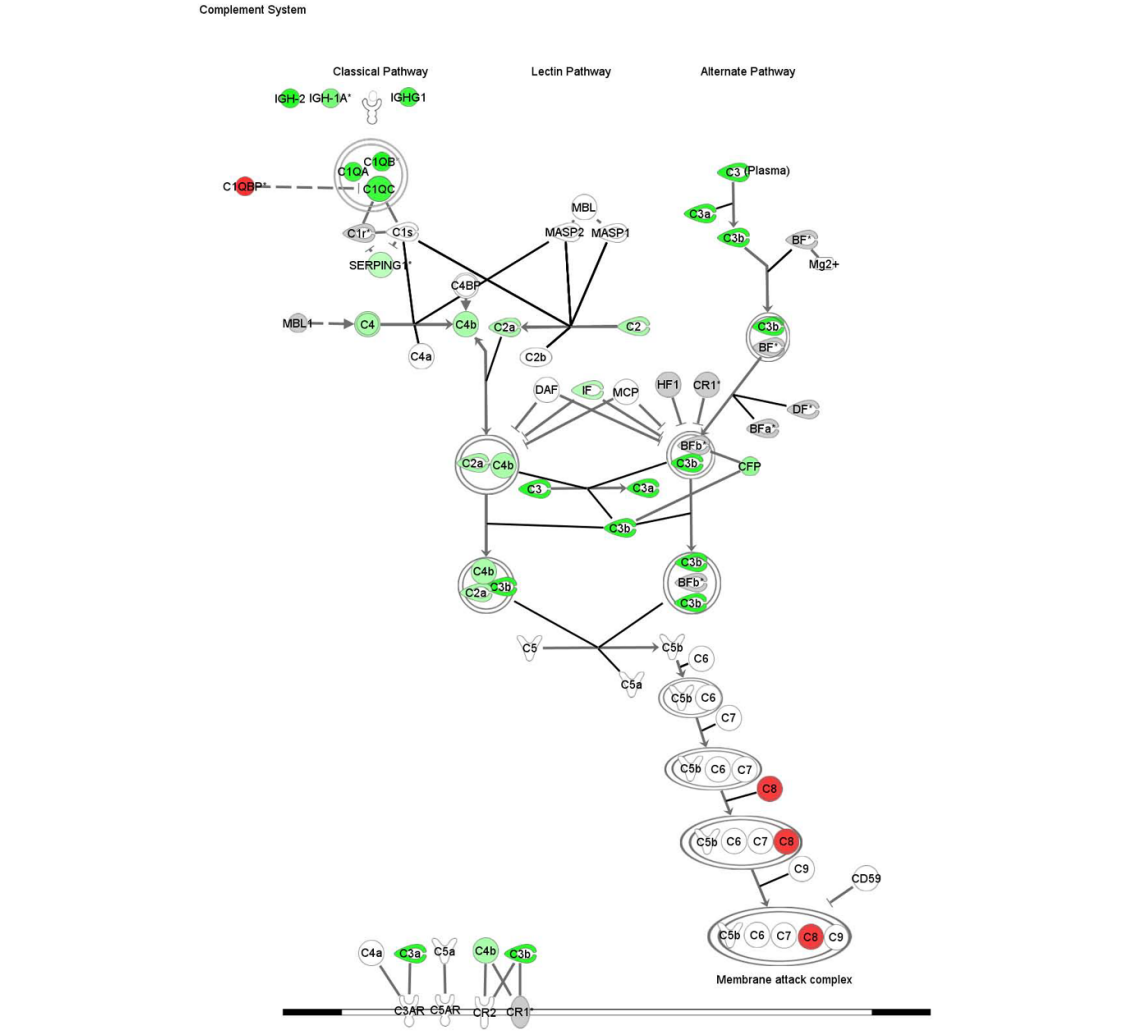
**Complement pathway altered in disease**. Expression of components of the complement pathway in nephritis compared with asymptomatic tissue. Green colour indicated increased expression and red colour indicates a decreased expression in nephritis tissue. Grey colour indicates that the fold change and FDR p < 0.05 or the sigPathway FDR p < 0.05 filters were not met. The names of the genes are in "nodes". The shape of the nodes is related to gene function/family membership. The lines between the nodes represent the connectivity between genes. Solid lines indicate direct protein-protein interactions. Dotted lines represent indirect interactions.

**Figure 10:**
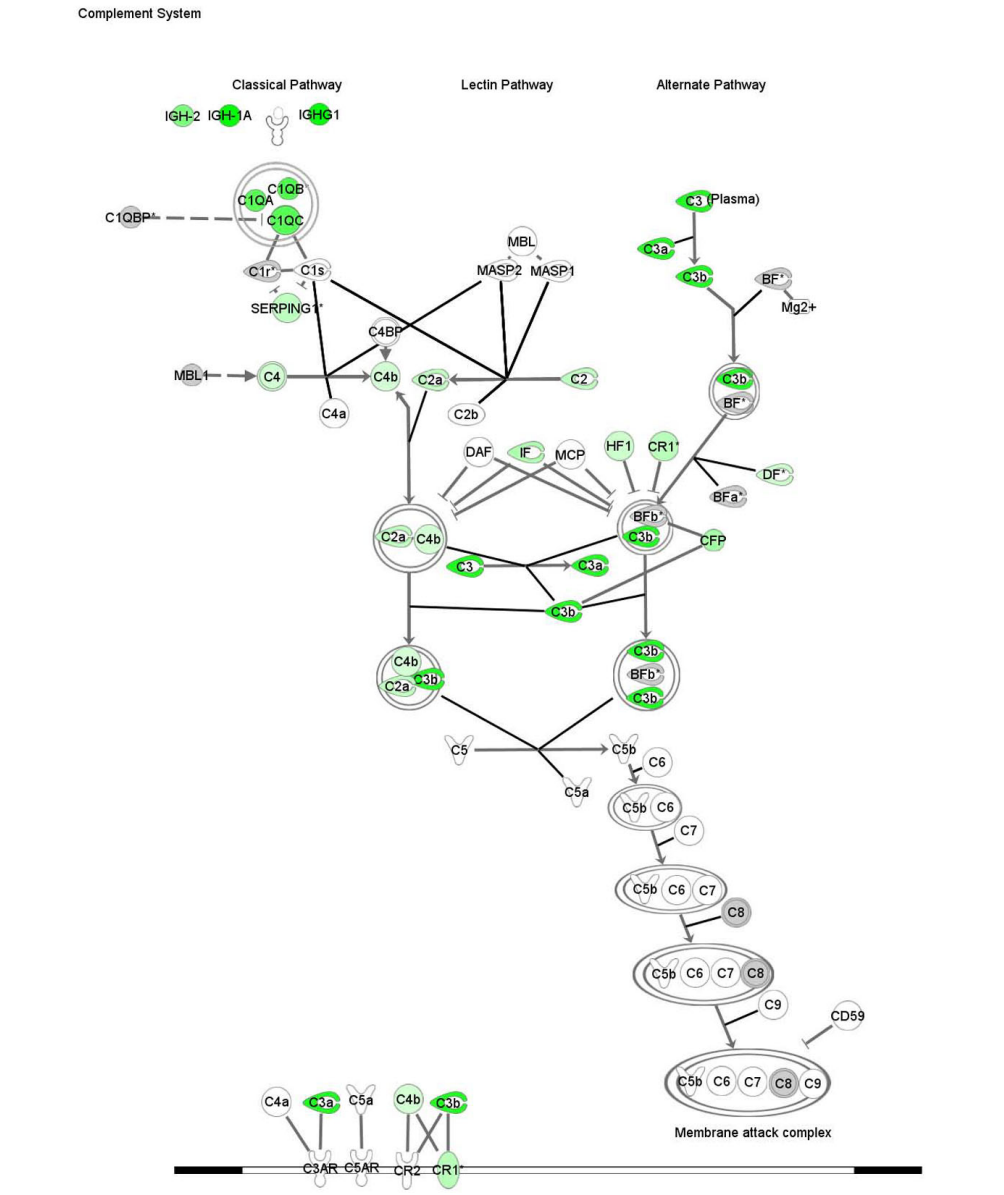
**Complement pathway modulated by sirolimus treatment**. Expression of components of the complement pathway in sirolimus-treated tissue compared with asymptomatic tissue. Green colour indicates increased expression and red colour indicates decreased expression. Grey colour indicates that the fold change and FDR p < 0.05 or the sigPathway FDR p < 0.05 filters were not met. The names of the genes are in "nodes". The shape of the nodes is related to gene function/family membership. The lines between the nodes represent the connectivity between genes. Solid lines indicate direct protein-protein interactions. Dotted lines represent indirect interactions.

Transcriptional analysis of kidney tissue in this model of nephritis generated three gene signatures for biological pathway comparison: disease-associated genes (identified by comparison of disease versus asymptomatic cohorts), sirolimus-responsive disease-associated genes (identified by comparison of diseased (control) versus sirolimus-treated cohorts), and sirolimus non-responsive disease associated genes (identified by comparison sirolimus-treated cohorts versus asymptomatic). Using the SigPathway algorithm, apoptotic gene sets and several mitochondrial gene sets (mitochondrial inner membrane and inner membrane proteins) were identified as being significantly associated with disease. Mitochondrial regulation of apoptosis was evident from these various gene sets, and this process is depicted in Figure 6. Sirolimus treatment restores the expression level of these gene sets to the asymptomatic levels, rendering this pathway insignificant (by SigPathway in the comparison of the sirolimus-treated and asymptomatic groups). Using a combination of SigPathway and/or IPA, other immuno-inflammatory networks linked to disease were identified. These included the antigen presentation pathway (Figures 7 and 8), complement pathway (Figures 9 and 10), and IL1 and IL10 signalling pathways (data not shown). Close examination of the antigen presentation pathway in the disease tissue identified elevated transcriptional expression of multiple components of the H2 locus involved in antigen presentation to both CD8+ and CD4+ T cells (Figure 7). A similar pattern is seen for these transcripts in the comparison of the disease and treated groups. The data show a treatment-dependent return to asymptomatic levels for some genes of this pathway, and a treatment dependent decrease below asymptomatic levels for some other genes (Figure 8). Evaluation of the complement pathway in the disease tissue shows increased transcriptional expression of key components of the classical pathway, C1qa, C1qb, C1qc, C4 and C3, the latter two are also components of the alternate pathway (Figure 9). Using SigPathway, additional members of the complement pathway C8, CFH and CFD were identified. Treatment with sirolimus returned the expression of the C3 and C1q to asymptomatic levels, while C4 in the classical pathway remained elevated (Figure 10 and data not shown).

A critical signalling pathway involved in mediating an inflammatory response is the JAK-STAT and MAP kinase pathway. Increased levels of transcripts for pathway members including JAK3, STAT3, SOCS3, PTPN1, CDKN1A, RRAS and MAPK1 were observed in nephritis. After treatment with sirolimus these pathways exhibit transcriptional expression levels similar to asymptomatic levels.

### Rapalog-mTOR canonical pathway and links to mouse lupus nephritis genes

Networks were built in an effort to understand the broad ranging beneficial effects of the mTOR pathway inhibitor, sirolimus, in NZB/W lupus nephritis. The first step in this process was to build a rapalog-mTOR pathway. This pathway consisted of the mTORC1 complex (mTOR, GBL, Raptor), the mTORC2 complex (mTOR, GBL, AVO3), the immediate downstream targets of mTOR – RPS6KB1 and RPS6KB2, and the upstream effectors of mTOR – AKT1, AKT2, TSC1, TSC2. In addition, rapalogs, such as sirolimus, temsirolimus and everolimus as well as all members of the immunophilin protein family that bind to the immunosuppressants FK506 and rapamycin, were included in the rapalog-mTOR pathway. Downstream connectivity of the rapalog-mTOR pathway to the 387 lupus nephritis genes was explored using the IPA. Of the 387 genes, 32 can be placed immediately downstream of the rapalog-mTOR pathway. An additional 25 of these are connected to the pathway through various types of functional protein interactions (eg, phosphorylation, binding etc).

**Figure 11:**
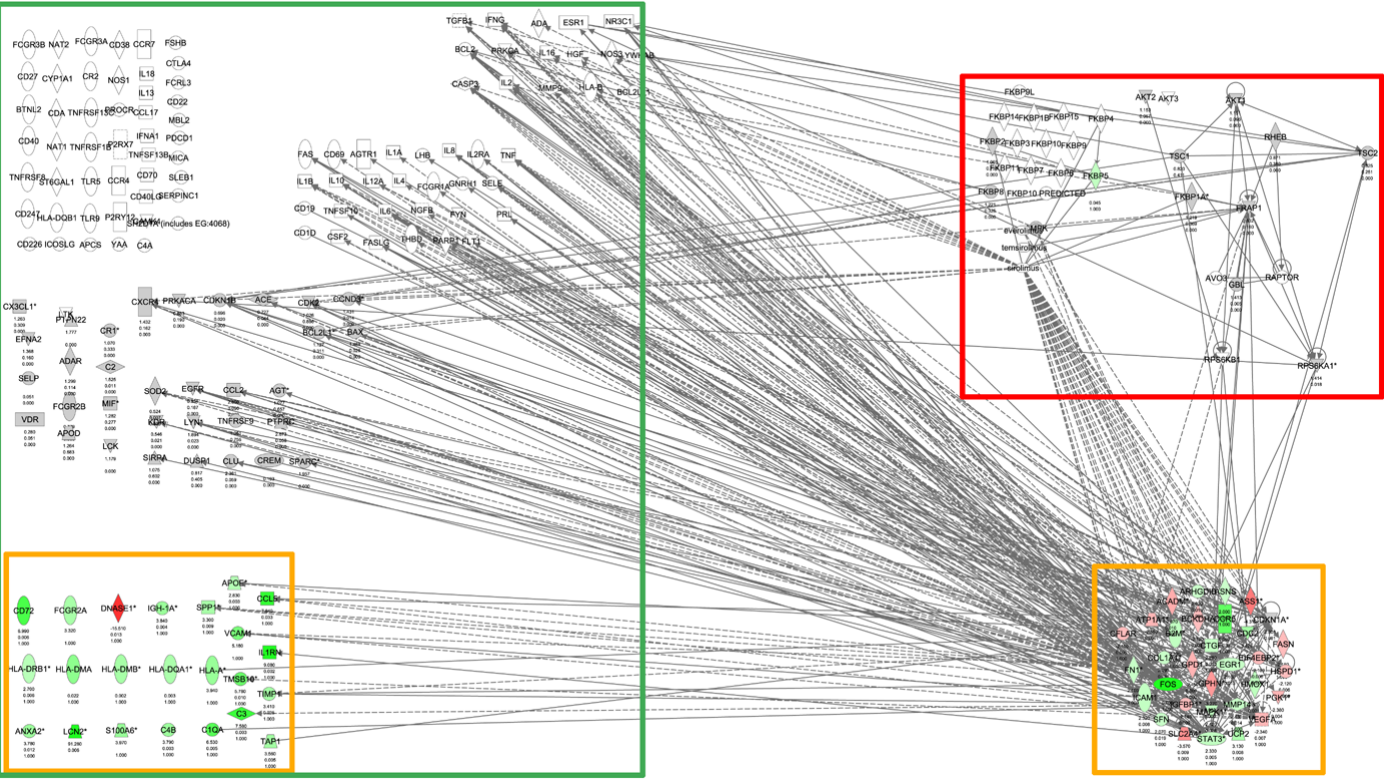
**Shortest path from the mTOR pathway to lupus nephritis genes and lupus genes**. The information shown schematically in this figure can be viewed in table form in Additional file 4. Solid lines indicate direct interactions and dotted lines indicate indirect interactions. mTOR pathway is indicated in the red box, the lupus nephritis genes in the yellow box and the lupus genes in the green box. The green colour nodes indicate genes expressed at higher levels in disease and red indicates lower levels in disease. Grey colour indicates that the fold change and FDR p < 0.05 filters were not met. The names of the genes are in "nodes". The shape of the nodes is related to gene function/family membership. The lines between the nodes represent the connectivity between genes. Solid lines indicate direct protein-protein interactions. Dotted lines represent indirect interactions.

Therefore, based on curated protein-protein and drug-protein interactions in the literature we determined that about 15% of the identified 387 nephritis genes interact with components of the rapalog-mTOR pathway. We therefore posed the question: Of the genes linked to lupus in the published literature, how many can be placed in the rapalog-mTOR pathway? Using curated findings in IPA, at least 50% of the known lupus-associated genes in IPA and Metacore interact with components of the rapalog-mTOR pathway. The large numbers of connections between the lupus nephritis genes we identified, the rapalog-mTOR pathway, and previously identified genes associated with lupus are shown schematically in Figure 11. The individual genes in each of these categories are listed in Additional file 4.

### Building the mTOR pathway interactome

All proteins known to interact with members of the rapalog-mTOR pathway, upstream and downstream were used to create the mTOR pathway interactome, consisting of 570 proteins (see Additional file 5). The magnitude of this 570 protein interactome is likely to be due in part to some high connectivity proteins such as the AKT gene group, which regulate many pathways. The 570 proteins from this interactome were imported into Metacore to query human diseases significantly associated with the mTOR pathway interactome. Significantly associated human diseases likely to be perturbed by mTOR pathway dysfunction were identified using the functional enrichment category within Metacore that relies on curated human disease specific networks.

### Human lupus and genes of the mTOR pathway interactome

Various cancer and non-cancer human diseases were identified as being significantly associated with the mTOR pathway interactome. A few cancers, such as ovarian neoplasm and pancreatic neoplasm, topped the list of associated diseases, and remarkably these were followed by lupus with significance of association p value of about E-8.5. Other non-cancer diseases that showed a less significant association are diabetes, obesity, Alzheimer's disease, multiple sclerosis and arthritis, all having an association of p < 0.01. Notably, the p values for the other pro-inflammation diseases were much less significant than those for lupus. For example, p values for both multiple sclerosis and arthritis are about 1E-3.

The genes in the mTOR pathway interactome that are associated with lupus are, in large part, distinct from the genes involved in the other non-cancer diseases. This is evident from the top scoring Gene Ontology cellular processes for each of these diseases. For example, in Metacore, the top scoring Gene Ontology cellular processes for lupus are related to apoptosis and cell death, while those for diabetes are related to carbohydrate metabolism, and those for obesity are related to fat metabolism. Both diabetes and obesity show a much more significant association with the mTOR interactome than any pro-inflammatory disease other than lupus.

## Discussion

NZB/W mice develop nephritis closely resembling that seen in human patients with lupus nephritis. Here we show that an eight-week course of 5 mg/kg sirolimus delivered three times a week starting at disease onset (about 25 weeks) and continuing for eight weeks markedly reduced symptoms of disease as measured by proteinuria, kidney histopathology and survival. By 52 weeks of age all treated mice were alive despite cessation of treatment many weeks earlier. Both results described here and previously reported studies [20,22,30], establish that sirolimus treatment prevents progression of autoimmune nephritis and prolongs survival in NZB/W mice. A comparable effect of sirolimus treatment has been observed in MRL/lpr mice, a second model of lupus and lupus nephritis [21].

We identified genes expressed at abnormal levels in NZB/W kidneys by comparing RNA levels in asymptomatic young mice and older mice with symptoms of lupus nephritis. These lupus nephritis genes were further shown to not be associated with the normal ageing process based on the observed differences between healthy young and old C57BL6 mice. A broad range of biological functions was represented among the lupus nephritis genes identified in this study. As expected, given the loss of kidney function, the vast majority of genes involved in metabolic pathways are down-regulated in nephritis and, given the inflammatory nature of the disease, many of the signalling pathway genes are up-regulated. Glomerular disease is a significant component in lupus nephritis. A recent study identified a glomerulus-enriched gene set [31]. We used data from this study to determine if the nephritis-associated genes are enriched in the glomerular gene set. We found a highly significant over-representation of the glomerular genes (chi-square value of 49.29 with one degree of freedom) consistent with glomerular involvement.

A recent study by Liu and colleagues reported on 126 nephritis-associated genes in the MRL/lpr model [17]. Of these, 37 genes were present in the nephritis-signature reported here. Commonalities were noted in the nephritis signatures (and this includes the directionality of change in the disease state) between these two models, such as the antigen presentation and complement pathways as well as various IFN-regulated genes and immunoglobulins. A good overlap (21 genes) was also noted between our mouse nephritis gene set and 68 human lupus nephritis genes derived from laser-captured glomeruli from SLE patients [32]. Additional similarities may be present, but probably lie outside the statistical parameters of both datasets.

A profound 'normalisation' of expression levels of lupus nephritis genes was observed in mice treated with sirolimus, both for metabolic as well as signalling pathways. Affected metabolic pathways in lupus nephritis include fatty acid degradation, glycolysis pathways and leucine/valine/isoleucine degradation. Transcripts for BCKDHA and DBT, two enzymes in the branched chain amino acid metabolism pathway required for the catabolism of leucine, valine and isoleucine, are reduced in nephritis, perhaps leading to the accumulation of leucine in diseased tissue. Interestingly, leucine activates the target of sirolimus inhibition, mTOR, leading to increased protein synthesis [33], and in addition we noted an increase in ribosomal RNA transcripts in the disease state. This physiological connection suggests that mTOR pathway activation may be increased by leucine in disease, providing perhaps an additional mechanism for sirolimus efficacy. Levels of these transcripts were returned to asymptomatic levels in sirolimus-treated mice. Several genes in the mitochondrial electron transport chain are also down-regulated in the disease state, and mitochondrial dysfunction has been implicated in kidney function impairment [34].

Reflecting the pro-inflammatory functions of nephritis, genes such as JAK3, STAT3 and MAPK1 involved in signalling pathways (JAK/STAT, MAP kinase, antigen presentation, IL1/IL10) are expressed at higher levels in the disease state. Also SOCS3, a negative regulator of JAKs and PTPN1 and CDKN1A, a negative regulator of STATs, are also elevated in the disease state. Although activation of these signalling pathways occurs through phosphorylation-dephosphorylation events of pathway components, it can be noted here that this pathway is also dysregulated at the transcriptional level in lupus nephritis. This complex dysregulation of the JAK/STAT pathway, which drives production of multiple cytokines and other inflammatory mediators, is returned to asymptomatic levels on sirolimus treatment. PTPN1, a negative regulator of STATs, is a notable exception, suggesting a link between the quiescence of this pathway with amelioration of disease. Consistent with the activation of this signalling pathway, genes involved in immune system cascades, such the IFN-regulated genes (IRF1, IRF7, OAS1, IFITM3, IFI27 and IGTP), and signalling by IL2 subfamily of type 1 cytokines (IL2Rγ) were also up-regulated in the disease state and are down-regulated by sirolimus.

Genes of the complement pathway known to be involved in renal damage, such as C3, C4, C1QA, CCL13 and FCGR2a, are also expressed at higher levels than in the untreated group. C3, C4 and C1QA play a role in antigen clearance. Using sigPathway [29], an algorithm that identifies differentially expressed gene sets, additional components of the complement pathway are transcriptionally elevated in the diseased renal tissue. Our results suggest that the complement components in the early parts of both the classical and alternate pathways are elevated in nephritis, while one component of the membrane attack complex, further downstream in the complement pathway, is down-regulated. C1q and C3, but not C4, were normalised by treatment. Complement pathway components are known to be significant contributors to renal damage. C3 deposition in the kidney has been observed in both human lupus nephritis and in murine models [35]. The elevated levels of C4 during disease amelioration is consistent with the concept that the early members of the classical pathway may be important in reducing disease pathology by clearing immune complexes and apoptotic cells [35,36]. Our profiling analysis also identified a large number of immunoglobulin transcripts elevated in the kidney tissue consistent with the role of autoantibodies and immune complex deposition in pathology.

To understand the mechanism by which sirolimus normalised such a wide range of biological processes, networks were built around the nephritis genes and the rapalog-mTOR pathway. Using curated findings from the literature, the shortest path for about one sixth of the 387 nephritis genes was defined to be either 0 or one-step downstream of the rapalog-mTOR pathway. This suggests a close functional association of mTOR pathway with disease mechanisms. In the context of the findings reported here, it is worth noting that steroid and cyclophosphamide, known to ameliorate lupus, directly impact some components of the mTOR pathway (data not shown).

In additional to preventing nephritis, sirolimus also had striking effects on the anti-DNA antibody titres in mice with lupus [20-22,30], so we addressed the connectivity of genes linked to any form of lupus with the mTOR pathway. About 50% of the lupus genes curated as lupus disease genes from human and rodent species in Ingenuity and MetaCore can be linked to the rapalog-mTOR pathway. The connectivity would, no doubt have been higher with the use of automatically extracted relationships from the biomedical literature. However, the algorithms used in automatic extractions cannot approximate human reasoning and return a mixture of true and false positives. Therefore, we relied exclusively on manually curated databases of protein findings and our results should be viewed as a lower estimate of connectivity.

To assess the significance of the association between human lupus genes and the mTOR pathway, we built an mTOR pathway interactome (ie, a network consisting of proteins that interact with the mTOR pathway) using IPA. We then queried which human disease networks (manually curated) in Metacore were best represented in the mTOR pathway interactome. Of the 87 human disease networks represented in Metacore, human lupus was identified as being highly significant, with only two cancers showing more significant associations. Additional cancer and non-cancer diseases were also identified through this process, including Alzheimer's disease and other autoimmune diseases such as multiple sclerosis and arthritis. Indeed recent work has uncovered a strong link between the mTOR pathway, Treg function and autoimmunity. Rapamycin was shown to inhibit AKT-mediated repression of FOXP3 (and AKT is upstream of mTOR). FOXP3 is a critical player in Treg cell differentiation and maintenance and deficiency of FOXP3 in both humans and mice is associated with multi-organ autoimmunity and lymphoproliferative disorders [37-40].

Having investigated the human disease-mTOR pathway connectivity, we then widened our analysis by exploring the validity of the claim of connectivity by searching the literature for data showing the effects on rapalogs on these human diseases. By conducting these analyses independently of Metacore, we confirmed the relationship between the mTOR pathway and some human diseases, such as multiple sclerosis [41,42], diabetes [43-45], arthritis [46] and some cancers [47,48]. A search of the clinical trial database [49] reports ongoing clinical studies with rapalogs in a number of these diseases, and the analyses we present here support such studies. Indeed early clinical results on the effects of sirolimus treatment of lupus patients show promise. Nine SLE patients that had been treated unsuccessfully with other immunosuppressive drugs had significantly improved disease scores after sirolimus treatment (BILAG p = 0.0218, SELDAI p = 0.00002) [50], and another clinical study is in progress [51].

Our analyses indicate that the coverage of protein-protein interactions in curated databases such as Ingenuity and MetaCore is comparable with up-to-date text-mined content derived using MedScan, a data mining/natural language processing tool (Ariadne Inc., Rockville, MD). For example, Ingenuity has 80 and MetaCore has 65 proteins/complex/groups that interact with the mTOR protein and MedScan identifies 115 proteins that interact with the mTOR protein. This level of overlap indicates a comprehensive coverage in the databases used for these analyses.

## Conclusion

Given our results and the results of others showing that inhibition of the mTOR pathway prevents progression of lupus nephritis in various mice models, we reasoned that perturbations of the mTOR pathway can lead to the phenotype of lupus nephritis. We also assessed the involvement of the mTOR pathway in human lupus by building the mTOR pathway interactome (genes connected to the mTOR pathway) and using bioinformatic algorithms to determine the significance of the overlap between the mTOR interactome and the published findings on genes involved in human lupus. We found a highly significant overlap. We suggest a similar approach of assessing significance of overlap between genes linked to human diseases and networks controlling animal model perturbations can be useful in understanding the relevance of animal models and the exploration of new indications for established therapies.

## Abbreviations

ANOVA: analysis of variance; FDR: false discovery rate; H & E: haematoxylin & eosin; IFN: interferon; IPA: Ingenuity Pathway Analysis; IL: interleukin; mTOR: mammalian target of rapamycin; PAS: periodic acid-Schiff; SD: standard deviation; SLE: systemic lupus erythromatous.

## Competing interests

PSR, JPS, MJC, SJG, WL, SM and MOT are currently employed by Wyeth Research, a company with two approved mTOR inhibitors, one in transplantation and the other in renal cell carcinoma.

## Authors' contributions

HML performed the transcriptional profiling and took part in data analysis, and contributed to writing the manuscript. PSR performed the pathway analyses and co-wrote the later manuscript drafts. JPS and SJG designed and directed the studies on the effects of sirolimus in NZB/W mice. MJC carried out the experiments on survival and proteinuria in mice. EG was responsible for the histopathological evaluation of kidney tissue. SM and WL contributed to the mining of clinical databases and infrastructure for text mining focused on mTOR. AJD was the senior director of the project, participated in all data analyses and wrote the first draft of the manuscript. MOT directed the expression profiling experiments and data analyses, and co-wrote the later manuscript drafts.

## Supplementary Material

Additional file 1 A pdf file containing data from our laboratory confirming the published results of others on sirolimus-dependent decreases in anti-dsDNA titres. Click here for file

Additional file 2 A pdf file containing information about an unsupervised cluster showing relationships between mouse groups. Click here for file

Additional file 3 An excel file containing the FDR p values and fold change metrics for genes associated with lupus nephritis and the effect of sirolimus treatment. Click here for file

Additional file 4 An excel file containing a table that gives the information from Figure 11. Click here for file

Additional file 5 An excel files containing a list of 570 proteins in the mTOR interactome. All proteins known to interact with members of the rapalog-mTOR l pathway, upstream and downstream were used to create this mTOR pathway interactome. Click here for file
