# Supplementary material for: Mapping similarities in mTOR pathway perturbations in mouse lupus nephritis models and human lupus nephritis
Source: Arthritis Res Ther. 2008 Nov 3;10(6):R127. doi: 10.1186/ar2541 (PMC2656226; doi:10.1186/ar2541)
Supplement: Additional file 1 — A pdf file containing data from our laboratory confirming the published results of others on sirolimus-dependent decreases in anti-dsDNA titres. [file ar2541-S1.pdf]

SLE-5:
Mean Serum Anti-nDNA Antibody Isotypes 10/04/99 (36 Weeks)

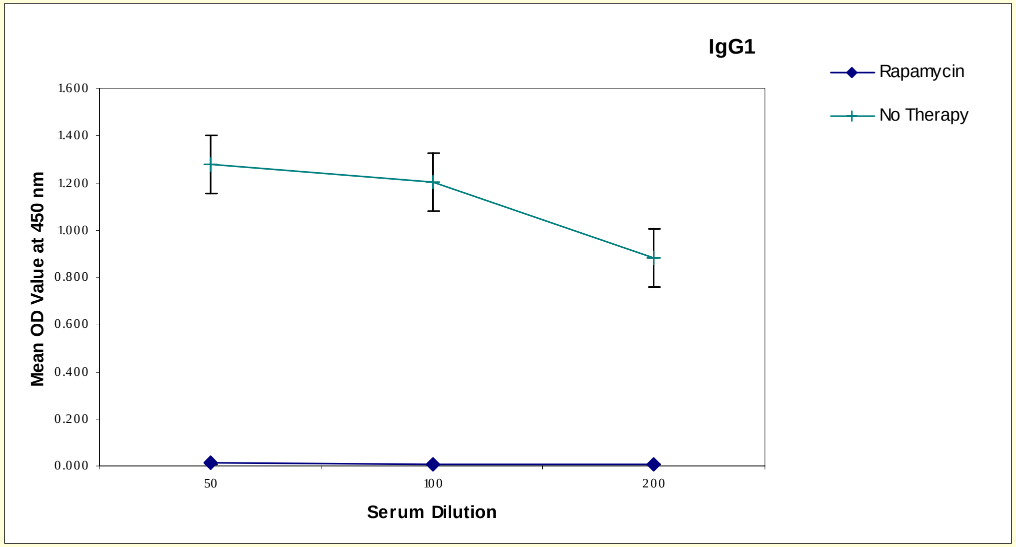

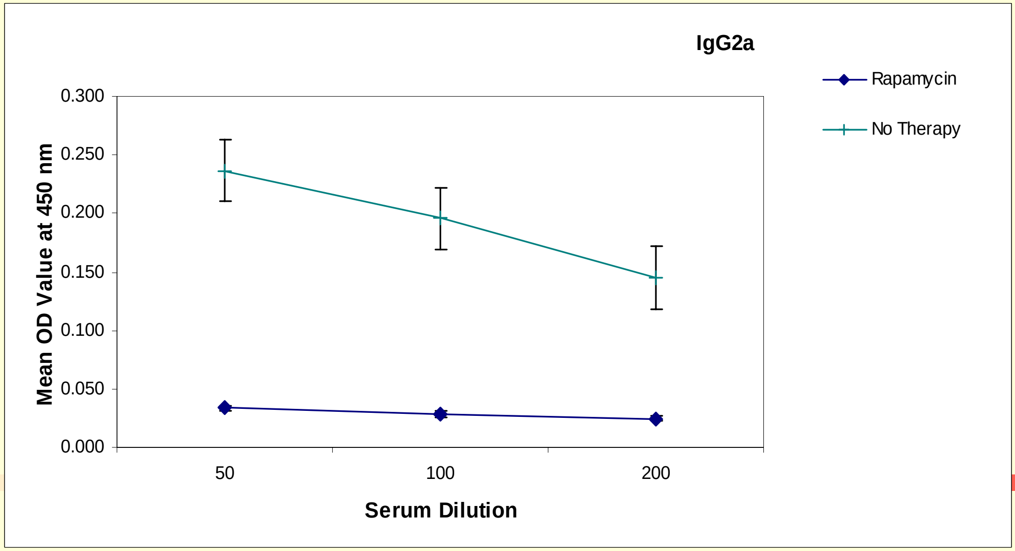

Wyeth Research

SLE-5:
Mean Serum Anti-nDNA Antibody Isotypes 11/05/99 (40 Weeks)

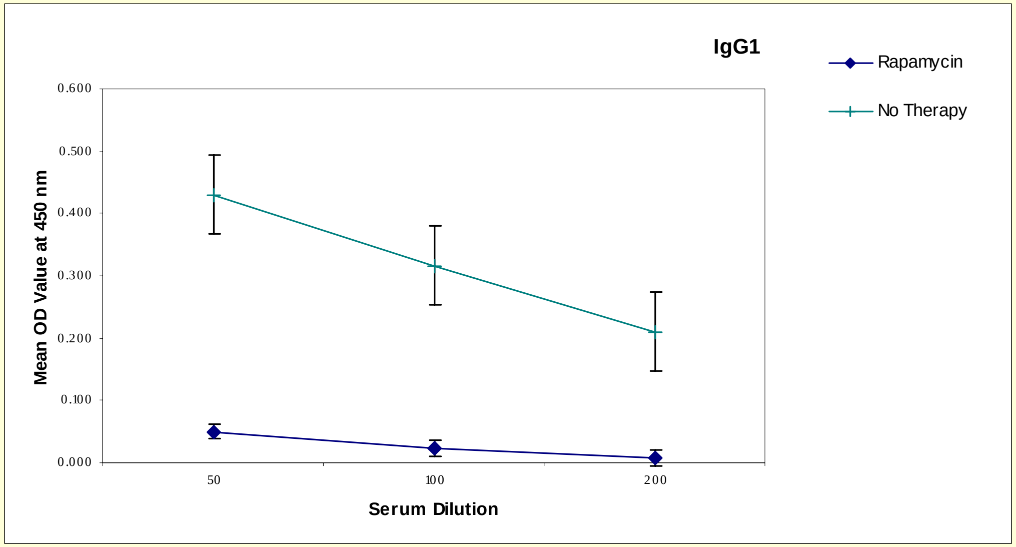

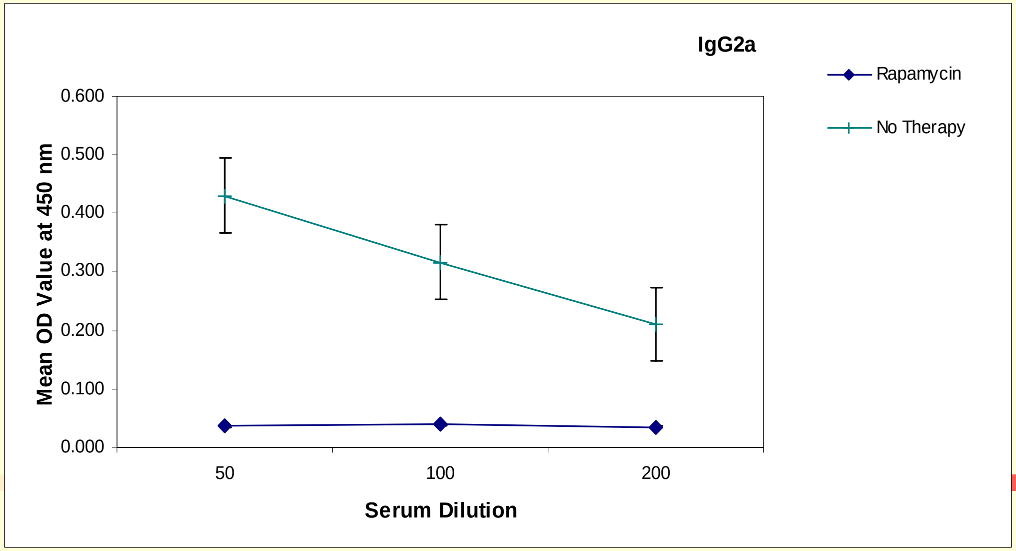

Wyeth Research

SLE-5:
Mean Serum Anti-nDNA Antibody Isotypes 12/30/99 (48 Weeks)

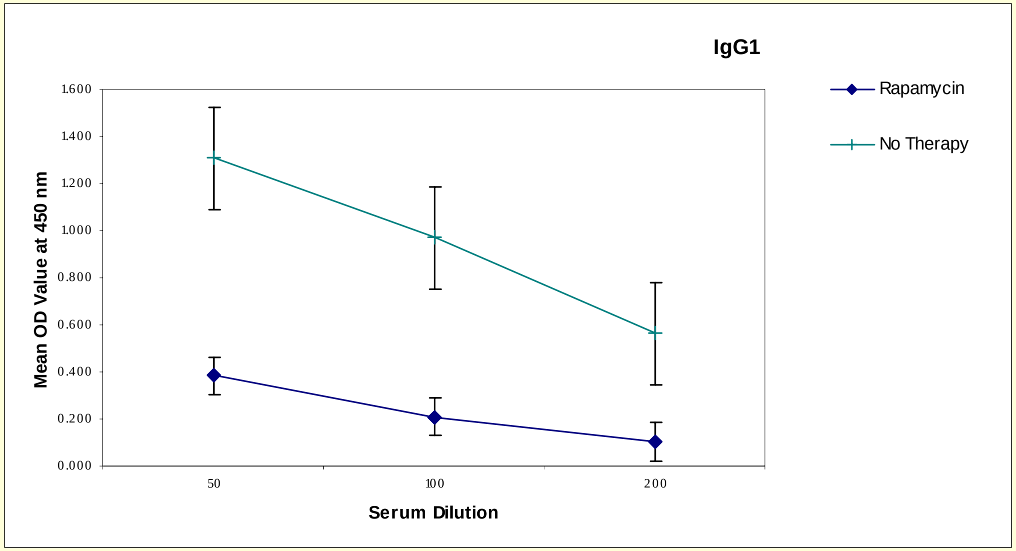

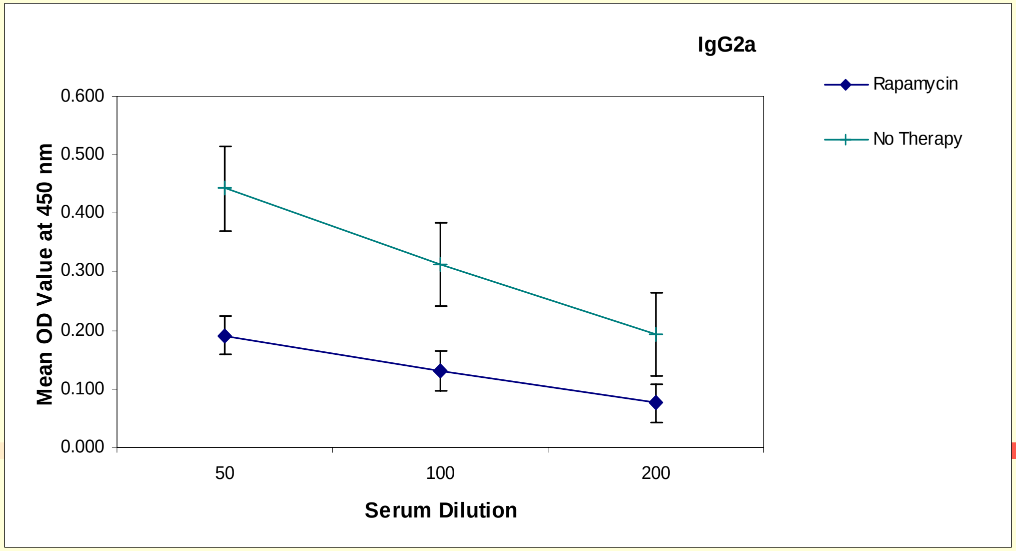

Wyeth Research
