# Supplementary material for: Mapping similarities in mTOR pathway perturbations in mouse lupus nephritis models and human lupus nephritis
Source: Arthritis Res Ther. 2008 Nov 3;10(6):R127. doi: 10.1186/ar2541 (PMC2656226; doi:10.1186/ar2541)
Supplement: Additional file 2 — A pdf file containing information about an unsupervised cluster showing relationships between mouse groups. [file ar2541-S2.pdf]

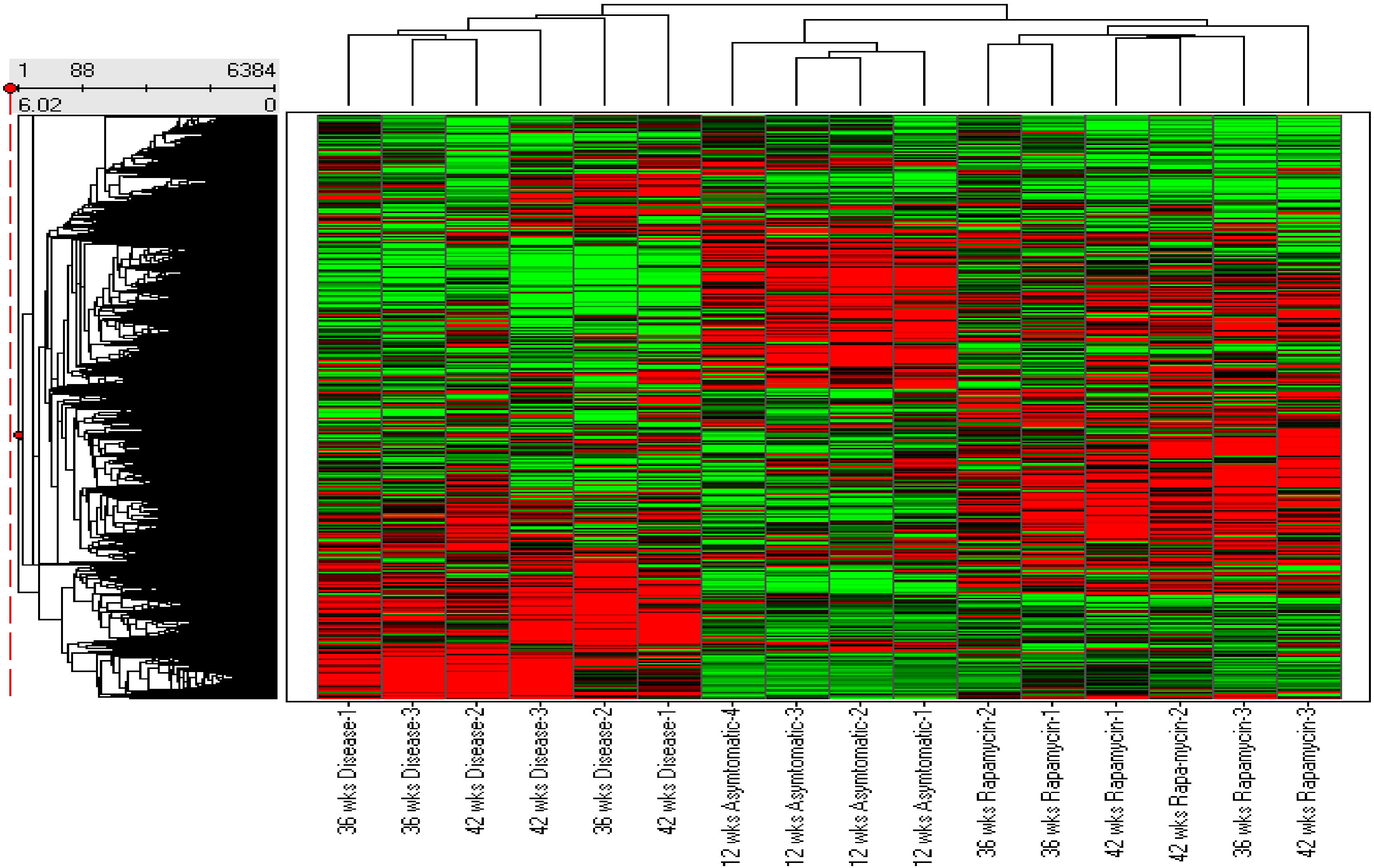

1
88
6384
6.02
0
36 wks Disease-1
36 wks Disease-3
42 wks Disease-2
42 wks Disease-3
36 wks Disease-2
42 wks Disease-1
12 wks Asymtomatic-4
12 wks Asymtomatic-3
12 wks Asymtomatic-2
12 wks Asymtomatic-1
36 wks Rapamycin-2
36 wks Rapamycin-1
42 wks Rapamycin-1
42 wks Rapa-mycin-2
36 wks Rapamycin-3
42 wks Rapamycin-3

Legend: Unsupervised cluster showing relationships between cohorts. Legend: Visualization of all 6387 probe sets that cluster the murine kidney sample time points. Subjects are shown in columns, and genes in rows. Red indicates a signal higher than the mean of all samples in the analysis. Green indicates a signal lower than the mean. Unsupervised clustering algorithm, which determines similarities between subjects independent of group membership, was used to generate this visualization. Subjects are grouped according to the degree of similarity in expression pattern. Note that the 36 and 42 week diseased murine kidney samples are grouped together, and that 12 week asymptomatic and 36 and 42 Sirolimus-treated samples cluster as individual subgroups on a separate node from the 36 and 42 week diseased samples. Every samples clusters to the appropriate group
